# Factors Associated With Hospital Commercial Negotiated Price for Magnetic Resonance Imaging of Brain

**DOI:** 10.1001/jamanetworkopen.2023.3875

**Published:** 2023-03-21

**Authors:** John Xuefeng Jiang, Ajay Malhotra, Ge Bai

**Affiliations:** 1 Broad College of Business, Michigan State University, East Lansing; 2 Division of Neuroradiology, Yale University School of Medicine, New Haven, Connecticut; 3 Johns Hopkins Carey Business School, Baltimore, Maryland; 4 Johns Hopkins Bloomberg School of Public Health, Baltimore, Maryland

## Abstract

This cross-sectional study investigates hospital characteristics associated with commercial negotiated price for magnetic resonance imaging of brain.

## Introduction

US hospitals are required by the Hospital Price Transparency rule to disclose pricing information for facility fees.^1^ Prior research documented widely dispersed commercial negotiated prices across hospitals for common shoppable radiology services (technical component only),^2^ which raised the question of what factors are associated with such price variations. In this study, we focused on a common and expensive radiology service: magnetic resonance imaging (MRI) of brain before and after contrast (*Current Procedural Terminology* code 70553; hereafter, *brain MRI*). We investigated hospital characteristics associated with commercial negotiated price nationwide and within the same state, hospital referral region, and health system.

## Methods

This cross-sectional study follows the STROBE reporting guideline for cross-sectional studies. This study did not fulfill criteria for human participants research in accordance with 45 CFR §46; therefore, institutional review board approval and informed consent were not required.

We obtained commercial negotiated prices and number of contracted plans for brain MRI for hospitals that complied with the Hospital Price Transparency rule as of June 13, 2022, from Turquoise Health,^3^ a data-platforming company whose compiled nationwide hospital price disclosure data were used in prior research.^2,4,5^ We excluded hospitals with missing values for these hospital characteristics (obtained from 2019 Medicare cost reports): ownership type, health system affiliation, rural location, teaching status, presence of salary expense for MRI department, number of beds, profit margin, charge markup, and payer mix, plus county poverty level (obtained from the 2020 Census Bureau).^6^ Variable definitions are listed in Table 1. The sample contained 2630 hospitals.

**Table 1.** Summary of Variables^a^

Variable	Definition	Hospitals, No.	Mean	Median (IQR)	Mean of nondisclosing hospitals (n = 1749)^b^	*P* value^c^
Commercial price, $^d^	The median of commercial negotiated prices within a hospital	2630	2268	1900 (1024 to 3197)	NA	NA
Nonprofit	1 if the ownership type is nonprofit; 0 if otherwise	2630	0.62	1 (0 to 1)	0.55	<.001
Government	1 if the ownership type is government; 0 if otherwise	2630	0.18	0 (0 to 0)	0.29	<.001
System	1 if system affiliated; 0 if otherwise	2630	0.70	1 (0 to 1)	0.47	<.001
Rural	1 if rural location; 0 if otherwise	2630	0.47	0 (0 to 1)	0.55	<.001
Teaching status	1 if teaching hospital; 0 if otherwise	2630	0.32	0 (0 to 1)	0.21	<.001
MRI salary	1 if has salary expense for MRI department (hiring clinicians); 0 if otherwise (outsourcing)	2630	0.54	1 (0 to 1)	0.37	<.001
Insurance plans, No.	No. of commercial health plans with negotiated prices	2630	16	11 (6 to 20)	NA	NA
Beds, No.	No. of available beds	2630	162	100 (25 to 230)	117	<.001
Profit margin, %	Net income/net patient revenue	2630	5.24	4.90 (−0.95 to 11.96)	3.15	<.001
Charge markup, %	Total charge/total Medicare-allowable cost	2630	438	373 (252 to 543)	342	<.001
Medicare proportion, %	Percentage of discharges covered by Medicare	2630	36.76	34.60 (26.09 to 44.91)	41.31	<.001
Medicaid proportion, %	Percentage of discharges covered by Medicaid	2630	7.91	4.36 (1.72 to 11.19)	8.50	.06
Poverty rate, %	Proportion of population in poverty in the county where a hospital is located	2630	12.54	12.00 (9.30 to 15.00)	13.39	<.001

Abbreviation: MRI, magnetic resonance imaging.

^a^ The sample contained 2630 hospitals that did not have missing values on any listed variable. Each variable was measured at the hospital level, with 2630 hospital observations. Poverty rate was obtained from the US Census Small Area Income and Poverty Estimates Program. Other variables were generated, as described in the definition column, from Medicare cost reports.

^b^ This column presents the mean value among 1749 hospitals that did not disclose pricing information and that had no missing value for each variable (except for commercial price and number of insurance plans).

^c^ *P* values were for 2-tailed *t* tests of the mean between disclosing and nondisclosing hospitals.

^d^ The national median Medicare rate is approximately $398.

We first compared characteristics between disclosing hospitals and nondisclosing hospitals. Next, consistent with prior literature, each hospital commercial price was measured as the median value of commercially negotiated prices.^2,4^ We used multivariable regression analyses including no fixed effects or state, hospital referral region, or health system fixed effects to identify factors associated with price variations at each level. Statistical analyses were conducted using SAS version 9.4 (SAS Institute) and Stata version 16 (StataCorp).

## Results

Price-disclosing hospitals were larger and more profitable and more likely to be nonprofit, system-affiliated, teaching hospitals, and located in urban areas and more affluent counties than nondisclosing hospitals. They had a higher charge markup, lower proportion of Medicare patients, and higher likelihood of employing clinicians in MRI departments (Table 1). These hospitals had a commercial price of $2268 (median [IQR], $1900 [$1024-$3197]) and contracted with 16 commercial plans (median [IQR], 11 [6-20] plans) for brain MRI. *R*^2^ information (Table 2) suggested that aside from variables included in the model, state, referral region, and health system characteristics explained price variation across hospitals by an additional 16%, 23%, and 36%, respectively.

**Table 2.** Regression Results for Factors Associated With Hospital Commercial Price for Brain MRI^a^

Factor	Commercial price, $							
	No fixed effects (n = 2630)		State fixed effects (n = 2630)		Region fixed effects^b^ (n = 2630)	Health system fixed effects (n = 2630)		
	Coefficient (95% CI)	*P* value	Coefficient (95% CI)	*P* value	Coefficient (95% CI)	*P* value	Coefficient (95% CI)	*P* value
Nonprofit (reference: for profit)^c^	277.8 (85.5 to 470.1)	.005	313.9 (125.9 to 501.9)	.001	386.5 (195.8 to 577.2)	<.001	NA	NA
Government (reference: for profit)^c^	300.3 (53.1 to 547.4)	.02	471.4 (219.3 to 723.5)	<.001	536.5 (276.4 to 796.6)	<.001	NA	NA
System (reference: independent)^c^	−75.0 (−217.5 to 67.5)	.30	−104.0 (−240.6 to 32.7)	.14	−112.6 (−258.2 to 33.0)	.13	NA	NA
Rural (reference: urban)	481.7 (339.6 to 623.9)	<.001	415.5 (279.6 to 551.4)	<.001	449.4 (307.8 to 591.0)	<.001	363.3 (156.8 to 569.7)	.001
Teaching status (reference: nonteaching)	−168.0 (−323.9 to −12.1)	.04	−144.9 (−293.2 to 3.4)	.06	−87.4 (−240.3 to 65.6)	.26	−85.7 (−307.6 to 136.3)	.45
MRI salary (reference: no MRI salary)	−18.4 (−143.0 to 106.1)	0.77	−1.4 (−121.4 to 118.5)	.98	17.2 (−110.2 to 144.5)	.79	−38.7 (−217.2 to 139.7)	.67
No. of insurance plans^d^	13.8 (9.9 to 17.7)	<.001	13.1 (9.1 to 17.1)	<.001	12.7 (8.5 to 16.8)	<.001	18.8 (9.1 to 28.5)	<.001
No. of beds^d^	−0.3 (−0.7 to 0.1)	0.15	−0.2 (−0.6 to 0.2)	.33	−0.1 (−0.5 to 0.3)	.55	−0.4 (−0.9 to 0.2)	.15
Profit margin^d^	11.8 (7.1 to 16.6)	<.001	6.4 (1.8 to 11.1)	.006	4.1 (−0.6 to 8.8)	.08	9.4 (2.4 to 16.3)	.009
Charge markup^d^	0.9 (0.5 to 1.2)	<.001	1.2 (0.8 to 1.6)	<.001	1.4 (1.0 to 1.8)	<.001	0.2 (−0.4 to 0.9)	.53
Medicare proportion^d^	13.5 (9.0 to 18.0)	<.001	15.9 (11.2 to 20.6)	<.001	13.6 (8.5 to 18.6)	<.001	15.3 (6.9 to 23.8)	<.001
Medicaid proportion^d^	15.2 (8.7 to 21.6)	<.001	17.3 (9.7 to 25.0)	<.001	12.9 (5.3 to 20.4)	.001	9.1 (−4.5 to 22.6)	.19
Poverty rate^d^	−24.6 (−37.4 to −11.8)	<.001	−0.9 (−15.8 to 13.9)	.90	−3.7 (−21.0 to 13.6)	.67	−2.8 (−26.2 to 20.7)	.84
Adjusted *R*^2^	0.09	NA	0.25	NA	0.31	NA	0.45	NA

Abbreviations: MRI, magnetic resonance imaging; NA, not applicable.

^a^ Estimated coefficients for the constant are not reported. Each variable had 2630 observations.

^b^ Hospital referral region information was obtained from Dartmouth Atlas of Health Care.

^c^ These variables do not vary within a health system and thus do not have estimated coefficients in column 4.

^d^ Coefficients are per 1-unit increase.

After controlling for various factors, nonprofit and government hospital commercial prices for brain MRI were higher than those of for-profit hospitals nationwide and within the same state or region (range across models, $277.8 [95% CI, $85.5-$470.1] to $386.5 [95% CI, $195.8-$577.2] and $300.3 [95% CI, $53.1-$547.4] to $536.5 [95% CI, $276.4-$796.6], respectively). Rural location, number of contracted insurance plans, and Medicare patient proportion were positively associated with commercial prices nationwide and within the same state, region, or health system (Table 2).

## Discussion

To our knowledge, this cross-sectional study is among the earliest studies that examined factors associated with hospital commercial negotiated price using self-disclosed price information in compliance with the Hospital Price Transparency rule. Nonprofit and government hospitals had higher commercial negotiated prices for brain MRI than for-profit hospitals. Across the nation and within the same state, referral region, or health system, hospitals located in rural areas, contracting with more health plans, or treating more Medicare patients had higher prices, potentially reflecting these hospitals’ stronger bargaining power compared with insurers in their local markets.

This study has several limitations. Results were confined to brain MRI and subject to omitted variable bias, estimation errors (time gaps among measuring variables), and data reporting and compilation inaccuracies. Moreover, due to the lack of information on the applicable time period of each hospital price, it is possible that some prices did not reflect actual prices at the time when data were collected. Importantly, this study was exposed to potential sample-selection biases because there was no pricing information from nondisclosing hospitals and hospitals with missing information on any variable included in the regression model analysis were excluded. These biases potentially affect the generalizability of results.

## Notes

Supplement.
Data Sharing Statement
