# Supplementary material for: Factors Associated With Hospital Commercial Negotiated Price for Magnetic Resonance Imaging of Brain
Source: JAMA Netw Open. 2023 Mar 21;6(3):e233875. doi: 10.1001/jamanetworkopen.2023.3875 (PMC10031386; doi:10.1001/jamanetworkopen.2023.3875)
Supplement: Supplement. — Data Sharing Statement [file jamanetwopen-e233875-s001.pdf]

Data Sharing Statement

Jiang. Factors associated with hospital commercial negotiated price for magnetic resonance imaging of brain. *JAMA Netw Open*. Published online March 21, 2023. doi:10.1001/jamanetworkopen.2023.3875

Data
Data available: No

Additional Information
Explanation for why data not available: The data were purchased from a commercial data provider.

© 2023 Jiang JX et al. *JAMA Network Open*.
